# Restraint Factors and Child Passenger Deaths in New South Wales, Australia

**DOI:** 10.3390/ijerph17041147

**Published:** 2020-02-12

**Authors:** Tom Whyte, Bianca Albanese, Jane Elkington, Lynne Bilston, Julie Brown

**Affiliations:** 1 Neuroscience Research Australia, Margarete Ainsworth Building, Barker St, Randwick, NSW 2031, Australia; 2 The George Institute for Global Health, Level 5, 1 King St, Newtown, NSW 2042, Australia; 3 Faculty of Medicine, University of New South Wales, Wallace Wurth Building, 18 High St, Kensington, NSW 2052, Australia

**Keywords:** child restraint, crash, injury, fatal, misuse

## Abstract

Inappropriate or incorrect use of child restraints can influence crash injury outcome. This study examined the role of restraint factors in child passenger deaths and the effect of legislation requiring appropriate restraint systems up to 7 years old. Data for child (0–12 years) passenger deaths occurring in New South Wales (NSW) from 2007 to 2016 were collected by the child death review team including photographs, reports of in-depth crash investigation, witness reports and medical reports. Restraint use, type of restraint, appropriateness of the restraint for the age of the child and correctness of restraint use were examined. The primary contributor to death was determined in each case. Sixty-four child passengers died in NSW during the data period. Twenty-nine (29/64, 45%) were properly restrained. Thirteen children (13/64, 20%) were unrestrained. In 20 cases (20/64, 31%), children were using a restraint that was either inappropriate for their age (6) or not used correctly (14). Restraint factors were a primary contributor in 22 (22/64, 34%) child deaths. Compared to pre-legislation, appropriate restraint use was more common post-legislation (13/22. 59% vs. 30/42, 71%). However, incorrect use was also greater (3/22, 14% vs. 11/42, 26%). Interventions targeting increasing restraint use and reduction of common ‘use’ errors are needed to prevent further restraint factor-related deaths.

## 1. Introduction

Injury is the leading cause of death and a major contributor to hospitalisations among children older than one year in Australia and developed countries [1,2,3]. A large proportion of these injuries are related to transport. Child passengers in motor vehicle crashes account for approximately 50% of transport-related injury in high-income countries [3].

The most effective way to reduce the risk of injury in a motor vehicle crash is to restrain the occupant in the vehicle [4,5,6]. For children to receive the maximum benefit from a restraint system, they need to be restrained optimally, which means using a restraint that is most appropriate for the child’s size and using the restraint correctly—exactly as intended by the manufacturer. Suboptimal restraint of children is associated with more serious injuries in the event of a crash [7,8] and has previously been reported to be common among both children admitted to hospital following a crash [7,8] and the general population of children travelling in cars [9].

Mandatory use of restraints by Australian children when travelling in vehicles was implemented in all states and territories by 1982 [10]. Laws required children under 12 months of age to be restrained by an Australian Standards approved child restraint and children over 12 months of age to be restrained by an approved child restraint or an adult seat belt. In 2010, new Australian road rules were implemented across Australia that specified use of age-appropriate restraints for children up to 7 years old. This legislation was individually adopted by each state and territory in Australia, and was enacted in the state of New South Wales (NSW). The legislation requires children up to at least the age of 6 months to be secured in a rearward-facing restraint, children aged from 6 months to 4 years secured in either a rearward- or forward-facing restraint with an inbuilt harness and children aged from 4 years to 7 years secured in a forward-facing restraint with an inbuilt harness or a booster seat [11].

In NSW, the child death review team (CDRT), convened by the NSW Ombudsman, maintains a register of child deaths [12]. The register contains data collected from public agencies such as the police who are legally required to provide the CDRT with full, unrestricted access to relevant records for the purpose of understanding the cause of the deaths to prevent and reduce future occurrences. Almost all vehicle crashes in NSW involving a fatality are examined in-depth by crash investigation and forensic police officers for law enforcement purposes. Fatal crashes involving child passengers are examined by the CDRT and hence this detailed level of crash information is routinely collected into the CDRT child death register. This provides unique data not typically available in other trauma databases to investigate crash and restraint factors contributing to child fatalities. The aim of this study was to use the in-depth detailed data provided in the CDRT dataset to examine the role of restraint factors such as non-use, inappropriate restraint for the child’s size and incorrect use of restraints in child passenger deaths in NSW over a ten-year period (2007–2016). A secondary aim was to explore differences in the restraint factor circumstances among child passengers fatally injured in crashes before and after the introduction of legislation requiring use of an appropriate child-restraint system by children up to the age of 7, in 2010.

## 2. Materials and Methods

This study is a single-case retrospective design and was approved by the University of New South Wales Human Research Ethics Committee (HC180293, approved 13 June 2018). To minimise the risk of identifying specific crashes or individuals, only group data is able to be reported. If group sizes are three or less, these are reported as three or fewer children to prevent the possible identification of individual cases.

Non-identifiable data collected for all child (0–12 years) deaths in passenger cars in NSW between 2007 and 2016 were provided by the NSW CDRT, summarising relevant crash, injury and restraint data. These data were primarily taken from photographs and reports of in-depth crash investigation and forensic police officers who attended the crash and/or inspected the site and vehicles involved, as well as witness reports and medical records including autopsy, where available.

Crash factors included in these data were the type of crash, type of vehicle(s) involved, impact opponent, type of impact, location of impact damage and road speed limit. Injury data included the cause of death and a detailed description of external and internal injuries for each body region. The restraint factors available included restraint use status, type of restraint used, appropriateness of the restraint type for the age of the child and the correctness of restraint use based on the definitions provided in Table 1. These restraint factors were classified from on-scene reports (by police, ambulance officers and witnesses) within the case file and corroborated by post-crash photographs and evidence of external injury.

**Figure 1:**
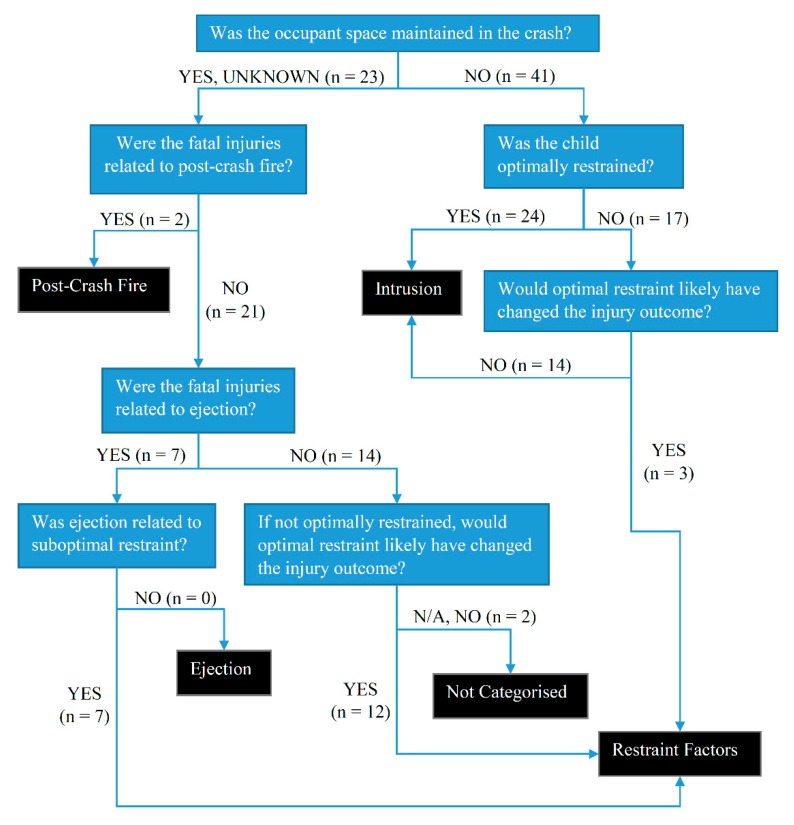
Flow chart for determining the primary contributor to death (black boxes) and the distribution of cases (in brackets).

The primary contributor to death in each case, defined as described in Table 1, was determined from the injury data, crash circumstances and restraint factors. Specifically, the mechanism and source of the fatal injury or injuries were determined in each case through assessment of injury data and crash factors. The flowchart shown in Figure 1 was used to categorize the primary contributor to death based on the available evidence in each case.

The annual rate of child deaths was calculated from the CDRT dataset and demographic statistics for NSW from the Australian Bureau of Statistics (ABS) [14]. The ABS data provided estimates of the resident populations of NSW based on the results of the 2011 Census of Population and Housing and the addition of population growth statistics. ABS data were extracted for each year corresponding to the CDRT dataset and was stratified by age to isolate those aged 0–6 years and 7–12 years.

The rate of child deaths and restraint factors (restraint status, restraint age appropriateness and correct use of restraints) were examined in the period 2007–2009 and compared to the period 2010–2016 to explore differences between the pre- and post- new legislation periods. Independent samples t-tests were run to examine whether there was any difference in the rate of child passenger deaths for age groups 0–6 years and 7–12 years before and after the 2010 legislation change.

## 3. Results

**Figure 2:**
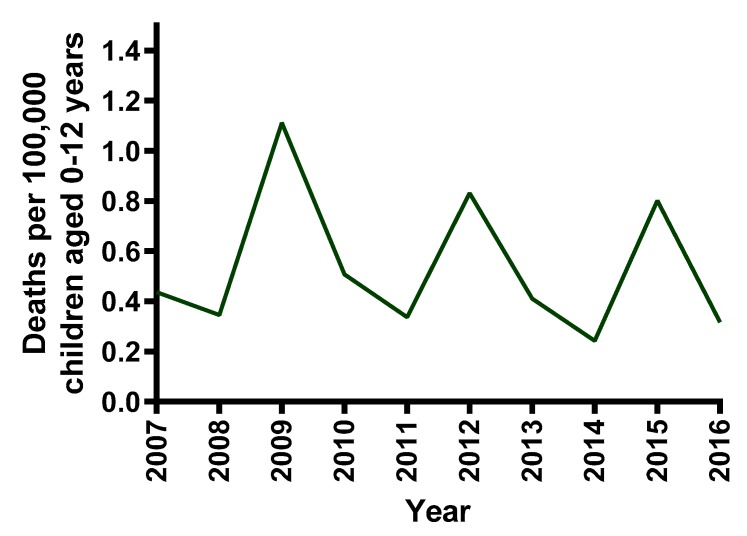
Rate of NSW child passenger (0–12 years) deaths per 100,000 children, 2007–2016.

In the ten-year period from 1 January 2007 to 31 December 2016, there were 64 children 0–12 years of age who died as passengers in cars in 60 crashes in NSW. The rate of child passenger deaths per 100,000 children is shown for each year in Figure 2. The sample consisted of 38/64 (59%) female and 26/64 (41%) male child fatalities. There were 37 children aged 0–6 years and 27 children aged 7–12 years.

### 3.1. Restraint Status

Of the 64 child passenger fatalities, 13 children (13/64, 20%) were unrestrained or likely to be unrestrained at the time of the crash. Hence, the majority of children who died (51/64, 80%) were using or likely using some form of restraint. However, only 29 of these children (29/64, 45%) were properly restrained. In 20 cases (20/64, 31%), children were using a restraint that was either inappropriate for their age (six cases) or was not used correctly (14 cases). There were two cases where there was insufficient information to determine whether the restraint used was age appropriate and/or correctly restrained.

Of the 13 children who were unrestrained or unlikely to be restrained at the time of the crash, nine were in the age group 0–6 years and four were in the age group 7–12 years. Nine of the 13 unrestrained children who died were ejected or partially ejected during the crash.

**Figure 3:**
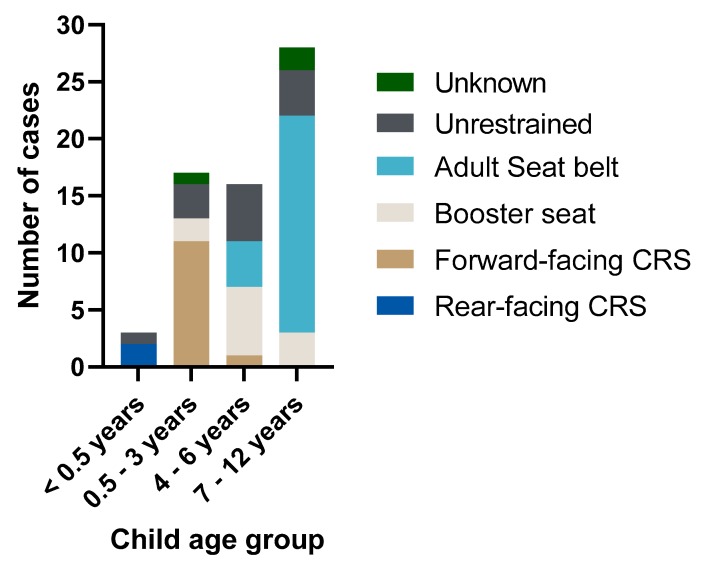
Restraint status for child age ranges in NSW child passenger deaths, 2007–2016.

Six children were using restraints that were inappropriate for their age and all were aged 2–6 years. Three or fewer children were using booster seats when they required a dedicated child-restraint system and four children were using adult seatbelts when they required a booster seat or child-restraint system. The restraint status of children in different age groups is shown in Figure 3.

Fourteen children were using age-appropriate restraints incorrectly. Incorrect use occurred in adult seat belts (three or fewer cases), booster seats (three or fewer cases) and forward-facing child restraints (eight cases). No incorrect use was identified in rearward-facing child-restraint systems.

Poor belt position (seat belts placed under the arm, children laying across the seat with the seat belt on) was the error in three or fewer adult seat belt misuse cases. In three or fewer booster seat misuse cases, poor belt position (poor routing of sash belt through booster seat) and top tether misuse were observed. For the eight forward-facing restraint misuse cases, inbuilt harness misuse (harness only partially used, harness positioned unevenly across the child, harness not properly adjusted for child height) was observed in six cases and top tether misuse (top tether not used, not anchored correctly, top tether loose or top tether twisted) was observed in four cases with three or fewer cases having both harness and top tether misuse.

### 3.2. Primary Contributor to Death

**Figure 4:**
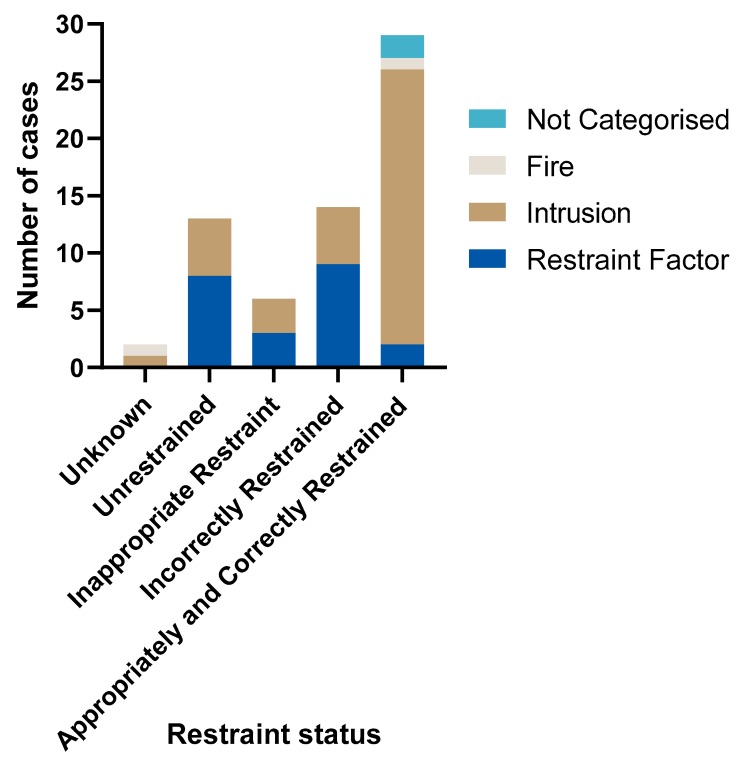
Primary contributor to death by restraint status.

Of the 33 children who were either unrestrained or not properly restrained at the time of the crash, the primary contributor to death was restraint factors in 20 cases (see Figure 4). The correct use of an age-appropriate restraint would not have prevented the deaths of the other 13 children who were either unrestrained or not properly restrained due to the nature and severity of the crashes, with factors such as intrusion and post-crash fire found to be the primary contributing factors in these fatalities.

Non-use of the restraint was found to be the primary contributor to death in eight of the 13 unrestrained children, with six of these having been ejected and three or fewer involved in avoidable fatal contact with the vehicle interior. Intrusion was the primary contributor in the remaining five unrestrained child occupant deaths.

Inappropriate restraint of a total of three or fewer children (three or fewer aged 2–3 years in a booster seat, rather than a child-restraint system, and three or fewer children aged 6 in an adult seat belt, rather than a booster seat), directly contributed to the injuries leading to death.

Of the 14 cases where the child was restrained incorrectly, correct use of the restraint may have prevented the deaths of nine of these children. Significant intrusion into the occupant space meant the outcome would not have been changed in five cases of incorrectly restrained children.

There were a further three or fewer cases among the 29 who were properly restrained according to NSW legislation where restraint factors were the primary contributor to death. These cases involved children using an adult lap belt-only restraint, rather than a lap-sash three-point restraint. Hence, restraint factors were a primary contributor to death in 22 (22/64, 34%) child deaths in NSW over the CDRT data period.

In the CDRT dataset, there was no evidence of any inadequacy in the performance of a child restraint when it was used correctly and appropriately (See Figure 4).

Overall, significant intrusion into the occupant space of the child was the primary contributor to death in 38 cases (38/64, 59%). In these cases, the events and/or severity of the crash meant that it was deemed unsurvivable even with optimal restraint practices.

Post-crash fire was the primary contributor to death in three or fewer cases.

Although ejection occurred in 11 cases, it was not the primary contributor to death in any case. In these 11 cases, restraint factors (seven cases of non-use of the restraint or misuse of the restraint) and significant intrusion into the occupant space during the crash (four cases) were the primary contributors to death. There were no cases of a child’s death due to ejection when the child was optimally restrained and the occupant space was maintained.

### 3.3. Rearward- or Forward-Facing Restraints for Young Children

For children aged 2 years and under, there were three or fewer children in rear-facing restraint systems, all under 6 months of age, and ten in forward-facing restraint systems, from as young as 8 months. For the rear-facing restraints, all children were correctly restrained. For the forward-facing restraints, three or fewer children were correctly restrained and seven children were incorrectly restrained with restraint factors being the primary contributor to death in four cases.

### 3.4. Differences in Restraint Factors Before and After Legislative Change

**Figure 5:**
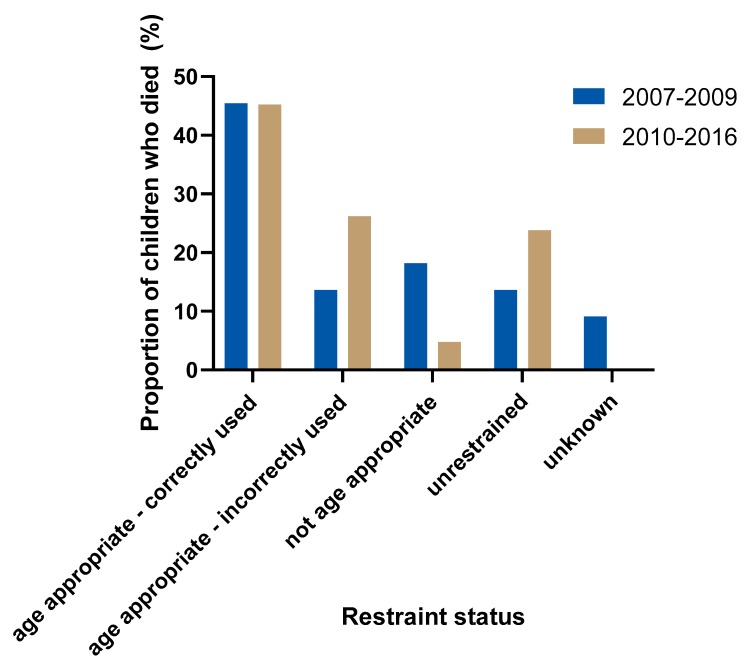
Restraint use status in NSW child passenger deaths pre- (2007–2009) and post- (2010–2016) child-restraint systems legislation change.

The proportion of age appropriateness and correct use of restraints in the period prior to the legislation change (2007–2009) is compared in Figure 5 to the period after the legislation was introduced requiring children up to age 7 to correctly use an age-appropriate restraint (2010–2016). The proportion of properly restrained children (using age-appropriate restraints correctly) who died in crashes remained the same (10/22, 45% vs. 19/42, 45%) in the period after the legislation change. The proportion of children using age-appropriate restraints increased from 59% (13/22) pre-legislation to 71% (30/42) post-legislation and the proportion of children using inappropriate restraints for their age decreased from 18% (4/22) to 5% (2/42). There was an increase in the proportion of fatally injured children who were using restraints that were not correctly used (3/22, 14% to 11/42, 26%) as well as an increase in the portion of those who were unrestrained (3/22, 14% to 10/42, 24%) following the change in legislation.

**Figure 6:**
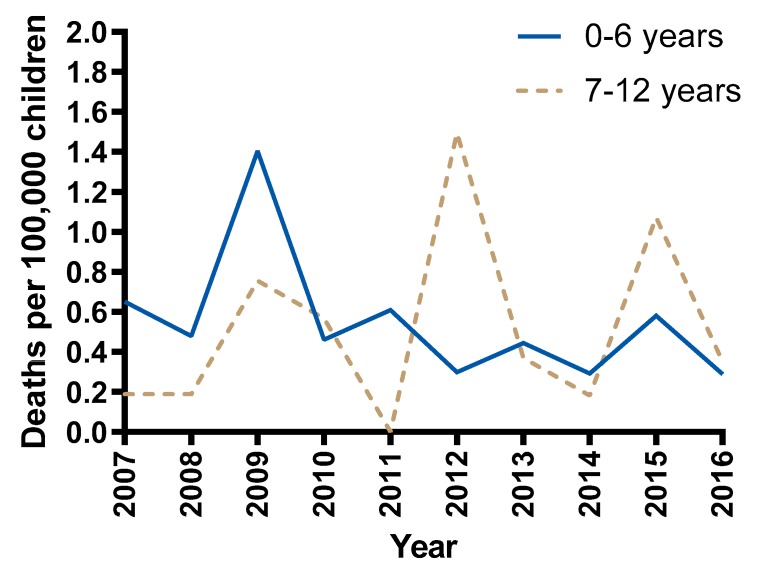
Rate of child passenger deaths in NSW 2007–2016 by age group.

The rate of NSW child passenger deaths per 100,000 children for two separate age groups over time is shown in Figure 6. The average rate of 0–6 year old child passenger deaths was not different in years 2007–2009 compared to years 2010–2016 (Welch’s t(2.133) = 1.45, *p* = 0.277), nor was the rate of 7–12 year old child passenger deaths between time periods (Welch’s t(6.311) = −0.722, *p* = 0.496). From 2010, the rate for children 0–6 years old has remained less than 0.6 deaths per 100,000 children, while for older children aged 7–12 the rate peaked at 1.5 and 1.1 deaths per 100,000 children in 2012 and 2015 respectively.

## 4. Discussion

The overall rate of child deaths while travelling in passenger cars in NSW over the period 2007–2016 demonstrates a slight but non-significant downward trend and regular year to year fluctuations. Given the relatively small numbers of child passenger deaths each year, which ranged from 3 to 13, it is difficult to attribute these fluctuations to any specific factor. However, this highlights the need to examine trends over time rather than comparisons between one single year and another. While there was no significant difference in the rate of passenger deaths after the introduction of legislation change requiring children under 7 years to travel in an age-appropriate restraint, the rate of child deaths per 100,000 population remained at 0.6 or below for 0–6 year old children and annual fluctuations have been less substantial than that observed for children aged 7 and older since the introduction of the legislation.

While in most cases the correct use of an age-appropriate standards-approved child-restraint system would not have altered the fatal outcome of the crash due to factors such as significant intrusion into the occupant space or a post-crash fire, in 34% (22/64) of cases restraint factors were the primary contributor to death suggesting increases in restraint use, use of appropriate restraints and correct use may have prevented these deaths.

The results of this NSW case series are similar to a set of 92 fatally injured children in the United States (US) in 2000. In their data, half of the crashes were considered unsurvivable and 12% judged to result from gross misuse of the restraint [15]. The larger proportion of unsurvivable cases in the current study (42/64, 66%) may reflect differences in contributing factor categories. In the US dataset, complete destruction of the child’s survival space was necessary to be deemed unsurvivable whereas significant intrusion at the child’s seating position was categorized as a non-catastrophic crash, circumstances that may have been categorized as unsurvivable intrusion in this study. The larger proportion of cases with restraint factors as the primary contributing factor to death in the NSW case series (22/64, 34%) is likely due to a wider and more comprehensive definition of inappropriate and incorrect restraint use than the US case series. In the US dataset, clear evidence of significant misuse was required for this classification (e.g., restraint not attached, or very loosely attached or internal harness not used).

### 4.1. Non-Use of Restraints

In 20% of child passenger deaths (13/64) in NSW between 2007 and 2016, the child passenger was unrestrained at the time of the crash. Based on the most recent population-level estimates of child restraint use indicating 0.8% of children travel unrestrained [9], restraint non-use is overrepresented in this sample of child passenger fatalities. High relative rates of restraint non-use are consistently reported among samples of fatally injured children [16,17] as well as adult drivers and occupants [18,19] indicating that being unrestrained is a major risk factor for fatal crash outcomes. A large proportion (9/13, 69%) of the unrestrained children who died in NSW reported in this study were ejected from the vehicle, which is known to increase the relative risk of fatal injuries compared to occupants who are not ejected [20]. Interventions targeting increased restraint use should be promoted in order to reduce the number of these child passenger deaths.

### 4.2. Inappropriate Restraints for Age

Across the sample, inappropriate restraint use for the age of the child based on current NSW legislation was observed in 9% (6/64) of child deaths in passenger cars. Inappropriate restraint use occurred only in children aged 2 to 6 years and was more common prior to 2010 and the introduction of the current legislation (Figure 5). Among children who died in NSW after 2010, only 5% were using an inappropriate restraint for their age. The proportion of inappropriate restraint use in this sample is considerably lower than previously reported in a cohort of crash involved children (71% of 2–4-year-olds, 68% of 5–6-year-olds) [7] and population-level estimates (51.2%) [9]. The greater proportion of unrestrained child passengers in the CDRT data influences the proportion of restrained occupants (age appropriate or otherwise) but the discrepancy may also reflect differences in the way appropriate use was defined in earlier studies and this study. In this study, appropriate use was entirely based on the age of the child, whereas earlier studies examined age along with other factors including height, weight and design specifications of different restraint types. The legislation introduced in 2010 requiring age-appropriate restraint use up to 7 years of age is a minimum requirement, and best practice advice is that children continue to use booster seats until they achieve an adequate fit from the seat belt [21]. No individual assessment of the adequacy of the seat belt for the child deaths in this study was possible due to the inability to compare individual child anthropometry with the interior geometry of each vehicle, and the retrospective nature of the review. It is likely that at least some of the children older than 7 years may have been better protected had they used a booster to assist with good seat belt fit. Furthermore, a lap belt-only restraint was identified as the primary contributor to death in two cases in this study and classified as legally appropriate whereby such a restraint may have been deemed inappropriate using alternative definitions.

### 4.3. Incorrect Restraint Use

Restraints were used incorrectly in 22% (14/64) of the child passenger deaths in the sample. This rate is lower than previous statewide estimates which found 50% of children were incorrectly restrained and 38% in a serious manner [22]. Again, these proportions are distorted by the higher incidence of unrestrained child passengers in the CDRT data. Furthermore, identification of incorrect use retrospectively is difficult, and typically under detects the presence of this form of restraint misuse. In this review, misuse was reported as being confirmed if police witnessed and accurately described the misuse, and likely if misuse was described in the witness statements and/or obvious from evidence of seat belt or harness webbing loading on the child’s body in post mortem injury descriptions.

A previous study that examined the change in Australian legislation on appropriate and correct use of child restraints at childcare centres, kindergartens, community centres, hospitals and child expos found no significant difference in the proportion of misuse and/or inappropriate use of restraints post-legislation compared to pre-legislation [23]. Contrastingly, a comparison of cross-sectional studies conducted before and after the 2010 legislation change found that both appropriate and correct use increased post-legislation among children 2–5 years old in low socioeconomic areas, although incorrect use still exceeded 50% of observed cases post-legislation [24]. In the current study, there was an increase in the proportion of incorrect restraint use among fatally injured child passengers following the 2010 legislation change but an accompanying decrease in the use of inappropriate restraints. It could be that with the reduction of inappropriate restraint use, incorrect restraint use became a more prevalent factor in child passenger deaths post-legislation change. Also, since misuse has been shown to be associated with more serious injuries in a crash [8], cases of incorrect restraint use are more likely to appear in the CDRT dataset since all cases have a fatal outcome. Given the relatively small sample size in this study, the increased proportion of misuse post-legislation may only reflect the need to achieve much higher rates of correct use in the population to see a noticeable and sustained reduction in restraint factors contributing to child passenger deaths.

The types of misuse identified in the child deaths reported in this study are common errors observed in a previous population observational study [22], including misuse of the sash belt for children in booster seats and seat belts, and misuse of the inbuilt harness and top tether for child-restraint systems. A previous US-based field investigation of forward-facing child-restraint systems in side impact crashes found that of eight crashes that resulted in moderate or more severe injury, there were four cases of misuse that likely contributed to the injuries sustained [25]. The predominant types of misuse were looseness of the harness and incorrect harness routing which act to increase head excursion or head acceleration. Although a relatively small sample, this high proportion of misuse effecting injury outcome in US data corroborates the findings of this study that restraint factors related to incorrect restraint use can have consequences for the crash injury outcome.

The majority of forms of incorrect restraint use in this study were ‘use’ errors (sash belt and inbuilt harness misuse) rather than installation errors (top tether misuse) and are therefore unlikely to be influenced by countermeasures such as ISOFIX-compatible anchorages systems and restraint-fitting stations. There is an urgent need to identify and develop effective countermeasures for reducing these more common use errors in addition to solutions for addressing installation errors.

### 4.4. Rearward- or Forward-Facing Restraints for Young Children

When children should be able to use forward-facing restraints is often debated. Australian legislation requires children to use rearward-facing restraints to a minimum age of 6 months, but both in Australia and in the US best practice recommendations are that children remain rearward-facing for as long as they fit in their rearward-facing restraint. A4-type restraints specified by the Australian standard are suitable for children from birth to approximately 30 months of age. Since the prevalence of rearward- and forward-facing restraint usage is typically informed by legislation and/or best practice guidelines, gathering statistical evidence for specific age-based criteria for when children are best protected in a rearward-facing restraint is difficult [26] because of biases in the crash involvement in each condition. In the CDRT data analysed in this study, there were no children older than 6 months who died in rearward-facing restraints, though the incidence of children older than 6 months who continue to use rearward-facing restraints is not known. The primary contributor to death was found to be restraint factors in a similar amount of forward (4/10, 40%) and rearward (1/3, 33%) facing children 2 years and under who died in NSW.

Although only a small sample size, restraint factors were not the primary contributor to death in the three or fewer rearward-facing children while restraint factors were the primary contributor to death in four of 10 children seated in forward-facing restraints. An observational study of children travelling in cars in NSW found that while installation errors were equally as common in rearward-facing child restraints compared to forward-facing restraint, securing problems were less common in rearward-facing restraints [22]. A laboratory study has also shown that the dynamic performance of a rearward-facing child restraint is less effected by user error than a forward-facing restraint [27]. The observations from the CDRT data and these previous studies suggest that the consequence for crash injury outcome may differ by forward and rearward-facing restraint type.

### 4.5. Observed Differences Before and After Legislative Change

There was more age-appropriate restraint use among children who died after the introduction of the 2010 legislation but there was also more incorrect use of restraints. Previous research suggests child restraint misuse increases with restraint complexity from seat belts, to booster seats, to forward-facing seats and convertible restraints [22,28]. Hence, the increase in incorrect restraint practices for younger children may reflect the increased use of more complex child-restraint systems for younger children required by the new law as compared to booster seats and seat belts. It is also possible that police investigators have become more vigilant in looking for evidence of incorrect use over time as general awareness of this issue has increased.

### 4.6. Other Factors in Child Passenger Deaths

Overall, significant intrusion into the space occupied by the child passenger was the most common primary contributing factor to death in the dataset (38/64, 59%). Intrusion is the result of the vehicle losing structural integrity as the energy of the crash exceeds the strength of the vehicle frame. Strategies to reduce the energy of the crash and increase the strength of the vehicles may have altered the fatal outcome in these crashes. Furthermore, the median vehicle age in this study at the time of the crash was 10 years yet more modern vehicles have been shown to better manage energy in a crash [29]. Vehicle crash testing and consumer information programs should continue to demand better performance of vehicles in maintaining the survival space of occupants in crashes.

While a detailed analysis of road environment factors was not performed in each case, it was clear most crashes occurred outside major cities on high speed roads (>80 km/h) and many with a single lane of traffic in each direction without any physical separation between vehicles travelling in different directions. Best practice from a safe system perspective would limit speed to 70 km/h on two-way single carriageway roads [30].

Post-crash fire was the primary contributor to death in two cases over ten years. The extent of fire involvement in road fatalities across Australia is unknown. A Swedish study found fire was involved in approximately 5% of all deaths in passenger cars, similar to the proportion of child deaths we observed in NSW, and that in one-third of these cases, the traumatic injuries were otherwise survivable [31]. The presence of these deaths suggests improvements in vehicle design focusing on fire prevention should be considered.

### 4.7. Limitations

Key limitations of the methods in this study include that data analysis was retrospective—data collection was originally managed by other organisations such as the police and was not necessarily focused on the research questions of this study. As a result, identification of restraint factors could not always be confirmed and identification of misuse was sometimes limited, meaning the number of restraint factors cases observed in this study is conservative.

While this retrospective in-depth case series provides important insight into the role restraint and other factors have played in the death of child passengers in Australia, the small dataset limits the use of advanced statistical methods and this may impact observations presented regarding changes after the introduction of the legislation. No definitive conclusions regarding the effect of the 2010 legislation change can be drawn from this dataset. Various factors affect fluctuation in a time series and these factors can change over time meaning the effect of legislation change cannot be isolated in this data. Investigating current child-restraint practices and comparing to previous reports [7,9,22], would help ascertain whether the trends in inappropriate and incorrect restraint use noted in this set of fatal crashes has changed since the adoption of the new legislation.

Furthermore, there was a transition period of three months for the introduction of the legislation in NSW in 2010 in which time a driver not complying was not fined nor deducted demerit points. Given the short timeframe of the transition period and the limitations of the dataset, it is difficult to ascertain whether this transition period had any effect on the variation in child passenger death rate.

Due to ethics and privacy issues we are unable to present and discuss individual cases in detail. Finally, there was some subjectivity in determining the primary contributor to death in the assessment of the mechanism and source of the fatal injuries although this was informed by the collection of evidence in the case file and followed the flowchart in Figure 1.

## 5. Conclusions

Restraint factors continue to be common primary contributors to child passenger deaths, accounting for 34% (22/64) in NSW, Australia, over a ten-year period from 2007 to 2016. Interventions targeting increasing use of restraints and reduction of common errors in use were identified in this case series as most important for preventing restraint factor-related deaths. An increase in the proportion of age-appropriate restraint usage among fatally injured child passengers and an increase in the proportion of incorrect usage observed in the post legislative change period compared to the preceding period further highlights the need to promote correct restraint use for children travelling in vehicles. However, more sophisticated analysis using population-level data is required to definitively draw conclusions about the impact of legislation on restraint use generally. Analysis of the CDRT data formed recommendations to the local road authority to monitor current child-restraint practices in NSW and promote best practice for restraining children in vehicles to increase correct and age-appropriate restraint use.

## Figures and Tables

**Table 1:** Categories and definitions for restraint factors at the time of the crash and the primary contributor to death, reproduced from [13].

Categories	Definition
Restraint status	
Unrestrained	The evidence provided confirmed that the child was unrestrained or indicated that the child was in all probability unrestrained, at the time of the crash.
Restrained	The evidence provided confirmed that the child was in all probability in a restraint the time of the crash. This term was used regardless of if it was the correct restraint for their age according to the current NSW legislation, or whether it was being used correctly at the time (as per manufacturer’s instructions).
Restraint type	
Rearward-facing child restraint	A restraint for children from birth, with a built-in harness, where the child faces the rear of the car. Sometimes known as ‘baby capsule’, ‘infant restraint’ or ‘baby carrier’. Type A in the Australian Standard.
Forward child restraint	A child restraint with a built-in harness where the child faces the front of the car. Sometime known as ‘child safety seat’, ‘forward-facing restraint’. Type B in the Australian Standard.
Booster	A child restraint that boosts the child up and positions the adult lap sash belt properly over the hips and chest of the child. It can be a booster cushion’ (backless), or a high back booster seat. Sometimes known as: ‘Belt positioning booster’, ‘booster cushion’ Type E, F in the Australian Standard.
Adult lap belt	A seatbelt that has no sash or shoulder part and only restrains the hips. Sometimes known as: ‘2-point seatbelt’.
Adult seat belt	A seat belt in the car that has a part of the belt that goes across the lap as well as a part that goes over the shoulder. Sometimes known as: ‘Lap and shoulder belt’, ‘3 point seat belt’.
Restraint age appropriateness	
Inappropriate restraint	Child was in the wrong restraint type for their age according to current NSW legislation, e.g., in a forward-facing restraint under the age of 6 months, in a booster seat while under the age of 4 years, or in an adult seat belt only under the age of 7 years.
Appropriate restraint	Child was in a restraint type appropriate for their age according to current NSW legislation.
Correct use of restraints	
Incorrectly used	The evidence provided confirmed that the restraint being used by the child was not used correctly, or was in all probability not used correctly, at the time of the crash. For example: Adult seat belts—misplacement of the seat belt sash under the arm, or the child was lying across the seat with the seat belt on. Booster seats—incorrect position of the sash belt, poor routing of the sash belt under side wings, top tether anchored to the wrong point, very twisted or loose tether strap or non-use of the top tether. Forward-facing or rearward-facing child restraints—errors in top tether use (e.g., anchored to the wrong point, very twisted or loose tether strap), non-use of the top tether, non-use or partial use of the internal harness, poor adjustment of the harness height (e.g., harness threaded through shoulder slots that are too high for the child, or through uneven slots), or the seat belt anchoring the restraint to the vehicle seat being unbuckled.
Correctly used	Installation and use of the restraint was exactly as the manufacturer intended.
Primary contributor to death	
Ejection	The child was thrown from the car to outside the car due to the crash.
Fire/explosion	Post-crash fire or explosion that was due to the crash.
Restraint factors	Factors associated with the restraint including non-use of a restraint, the wrong type of type of restraint for the child’s age (as per NSW legislation), or misuse of the restraint in terms of not it not being installed correctly or the child not being secured in it correctly according to the manufacturers’ instructions.
Intrusion	An object protruding into the internal space of the car.
